# Microbiome Profiling Reveals a Microbial Dysbiosis During a Natural Outbreak of Tenacibaculosis (Yellow Mouth) in Atlantic Salmon

**DOI:** 10.3389/fmicb.2020.586387

**Published:** 2020-10-22

**Authors:** James W. Wynne, Krishna K. Thakur, Joel Slinger, Francisca Samsing, Barry Milligan, James F. F. Powell, Allison McKinnon, Omid Nekouei, Danielle New, Zina Richmond, Ian Gardner, Ahmed Siah

**Affiliations:** ^1^CSIRO Agriculture and Food, Hobart, TAS, Australia; ^2^Atlantic Veterinary College, University of Prince Edward Island, Charlottetown, PE, Canada; ^3^Institute of Marine and Antarctic Studies, University of Tasmania, Launceston, TAS, Australia; ^4^CERMAQ Canada, Campbell River, BC, Canada; ^5^British Columbia Centre for Aquatic Health Sciences, Campbell River, BC, Canada; ^6^Elanco Animal Health, Charlottetown, PE, Canada; ^7^Food and Agriculture Organization of the United Nations (FAO), Animal Health Service, Rome, Italy

**Keywords:** *Tenacibaculum maritimum*, microbiome, yellow mouth, aquaculture, dysbiosis

## Abstract

Tenacibaculosis remains a major health issue for a number of important aquaculture species globally. On the west coast of Canada, yellow mouth (YM) disease is responsible for significant economic loss to the Atlantic salmon industry. While *Tenacibaculum maritimum* is considered to be the primary agent of clinical YM, the impact of YM on the resident microbial community and their influence on the oral cavity is poorly understood. Using a 16s rRNA amplicon sequencing analysis, the present study demonstrates a significant dysbiosis and a reduction in diversity of the microbial community in the YM affected Atlantic salmon. The microbial community of YM affected fish was dominated by two amplicon sequence variants (ASVs) of *T. maritimum*, although other less abundant ASVs were also found. Interestingly clinically unaffected (healthy) and YM surviving fish also had a high relative abundance of *T. maritimum*, suggesting that the presence of *T. maritimum* is not solely responsible for YM. A statistically significant association was observed between the abundance of *T. maritimum* and increased abundance of *Vibrio* spp. within fish displaying clinical signs of YM. Findings from our study provide further evidence that YM is a complex multifactorial disease, characterized by a profound dysbiosis of the microbial community which is dominated by distinct ASVs of *T. maritimum*. Opportunistic taxa, including *Vibrio* spp., may also play a role in clinical disease progression.

## Introduction

Tenacibaculosis [yellow mouth (YM)] is an emerging disease in Western Canada, which causes significant outbreak events in post-seawater entry Atlantic salmon (*Salmo salar*) smolt (Avendano-Herrera et al., 2006b). In British Columbia (BC), this disease is characterized by distinctive yellow pigmented plaques within the oral cavity. The disease typically occurs following the transfer of smolt to sea cages. YM can result in up to 40% total cumulative mortalities in affected cages. Reported mortalities attributable to YM occur during periods of high salinity and seawater temperatures (Frisch et al., 2018a, b). In BC, the annual cost associated with outbreaks is estimated to be $1.8M, based on the 2016–2017 year-class generations. This is in addition to an estimated revenue loss of $3.8M per year to the local industry (Powell and Podlasly, 2015). The current YM mitigation strategy is antibiotic treatment during the first 2 months following seawater entry. Due to a lack of vaccines, the use of in-feed antibiotic (sulfonamides or florfenicol) is currently the only treatment of YM in BC and worldwide. Although antibiotic treatment is essential for fish welfare and farm productivity, the use of antibiotics in BC waters remains a challenge for salmon farming companies.

*Tenacibaculum maritimum* (formerly classified as *Flexibacter maritimus*) is a Gram-negative filamentous rod-shaped bacterium and has been shown to be the principal etiological agent of mouth rot (Frisch et al., 2018a). Phylogenetic analysis of *T. maritimum* isolates derived from BC demonstrate that they clustered into two different sequence types (STCan1, STCan2) (Frisch et al., 2018a). Genetically these BC isolates were closely related to the Norwegian Lumpfish (NLF-15) and Chilean Atlantic salmon strain (Ch-2402) types previously identified (Smage et al., 2016b; Apablaza et al., 2017; Frisch et al., 2018a). Collectively, these strains are classified among subgroup C based on the study by Habib et al. (2014).

Despite the increased research effort, several knowledge gaps exist concerning our understanding of the complex etiology and epidemiology of the YM disease, as well as the potential mitigation strategies to control outbreaks. As the etiological agent is not clearly defined for YM, the involvement of different *Tenacibaculum* species in the manifestation of the disease has been postulated (Fernandez-Alvarez and Santos, 2018). Furthermore, the role of non-Tenacibaculum species in clinical disease progression of YM also remains unclear. In BC, YM outbreaks are believed to be multifactorial and associated with various host (e.g., smolt size and quality) and environmental (e.g., temperature and salinity) factors.

Many aquatic animal diseases are now recognized to be associated with pronounced shifts in microbial community structures, which ultimately have a negative effect on the host (Egan and Gardiner, 2016); a phenomenon known as “dysbiosis.” Infectious diseases in the marine ecosystem are increasing in emergence, severity, and prevalence, and many are opportunistic or environmental. Deleterious changes in microbial community structure can be caused by environmental stressors, infections and disease, and/or therapeutic treatment applications (i.e., antibiotics). While, in the case of YM, *Tenacibaculum* spp. play an important role in disease etiology, it remains unclear whether the clinical signs associated with YM are influenced by more widespread dysbiosis of the oral cavity. We surmise that *Tenacibaculum* spp. are present on salmon farms as opportunistic bacteria, and that a complex (multifactorial) etiology is involved in YM outbreaks.

The present study aimed to investigate changes in the microbial community associated with a naturally occurring YM outbreak in Atlantic salmon. Using 16s rRNA amplicon sequencing, we examined clinically affected and apparently healthy fish during an outbreak of YM disease. Furthermore, using the 16s sequencing approach, our study also profiled the oral microbial community in the same Atlantic salmon cohort post the YM disease event which showed no clinical signs of disease. The post-YM samples, designated “survivors,” represent animals that either became infected with YM and recovered, or animals that remained unaffected throughout the disease outbreak.

## Materials and Methods

### Sample Collection

The microbial community was profiled from commercially farmed Atlantic salmon smolt during and after a natural outbreak of bacterial stomatitis (YM) from BC, Canada in 2018. Atlantic salmon smolt with an average weight of 101.8 g were stocked into sea pens on a commercial farm site on the west coast of Vancouver Island on the 28th of September 2018. Fish were feed a commercial pelleted diet following stocking. YM related mortalities were first observed on the 6th of October 2018, with the peak of mortalities occurring on the 17th of October. The average water temperature preceding the YM outbreak was 13.02°C. Pale yellow plaques characteristic of YM were clearly visible in the mouth cavities of dead fish. On the 12th of October, a random sample of 20 smolt, displaying gross clinical signs of YM, was sampled from two commercial pens (10 individuals from pen 101 and 10 individuals from pen 102). While the severity of lesions was not quantified in the present study, we recorded no observable difference in disease expression between the two pens. All 20 YM affected fish displayed gross clinical signs including multifocal raised yellow plaques in around the palate and teeth. Gross lesions were clearly visible to the naked eye. To obtain samples, the smolt were first anesthetized and their clinical signs recorded. A mucosal swab sample was then taken from the mouth cavity of each fish using a sterile cotton swab and immediately stored in RNAlater. An additional 20 smolt which showed no clinical signs of YM, designated “healthy,” were sampled on the same day using the methods described above. Due to logistical limitations, all healthy samples were taken from the single pen 101. Following the disease event when YM mortalities had ceased, a further 20 samples were collected from pen 101 on the 5th of November 2018 from apparently healthy “surviving” individuals from the specific net pen. These fish showed no gross clinical signs of pathology in the oral cavity. It is important to note that that clinical history of the individual surviving fish that were sampled was unknown. That is, we cannot confirm that these fish had previously developed YM and then recovered, or if they had remained healthy throughout the YM disease event. Furthermore, because these samples were taken approximately 3 weeks after the peak of the disease outbreak, temporal changes in the microbial community unrelated to YM may have occurred. Nevertheless, we believe the inclusion of these samples adds additional insight into the apparently “normal” oral microbial community at a time when YM mortalities were not occurring.

Water samples were also collected from plankton tows using a 0.2 μM sterivex filter pre-stocking (27th of September), during the YM outbreak (12th of October 2018), and following the disease event (22nd of May 2019). The three water samples were collected at the same location on each occasion (adjacent to pen 101). The sterivex filter was stored in RNAlater prior to DNA extraction.

### Nucleic Acid Extraction and 16s Sequencing

DNA was extracted from each swab sample using the DNeasy Blood and Tissue kit (Qiagen) exactly as per the manufacturer’s instructions. DNA was extracted from the sterivex filters using the PowerSterivex DNA kit (Qiagen) as per the manufacturer’s instructions. Eluted DNA from either swab or sterivex was quantified using a spectrophotometer. Prior to library construction, the exact quantity of DNA was determined using Picogreen fluorescence (Invitrogen). Microbial profiling was performed using 16s rRNA amplicon sequencing. Briefly, the V1-3 variable region of 16s was amplified using the 27F (Lane, 1991) and 519R (Lane et al., 1985) primers as previously described (Simon et al., 2020). Both 27F and 519R primers contained Truseq Illumina adapter sequences. The PCR was performed in a 25 μl reaction containing 1X KAPA HiFi HotStart Ready mix (Roche), 0.3 μl of 27F and 519R primers, and approximately 10 ng of genomic DNA. The PCR was subjected to the following thermal cycling: 95°C for 3 min, followed by 25 cycles of 95°C for 30 s, 55°C for 30 s, and 72°C for 30 s. A final elongation at 72°C for 5 min was included. Amplification was confirmed by running 1 μl of the PCR was on a Bioanalyzer DNA 1000 chip (Agilent). An amplicon of between 500 and 600 bp was observed in all samples. This product was sequenced using 300 bp paired end chemistry on the Illumina MiSeq platform (Illumina).

### Bioinformatics

For each sample, the paired raw sequence reads were first merged using Flash (ver 2.2) with a minimum overlap of 30 bp and maximum overlap of 250 bp. The numbers of raw and merged reads are presented as Supplementary Table 1. The 27F and 519R primer sequences were stripped from the merged reads using a 20 bp left and 18 bp right truncation using Usearch (32-bit, ver 11). The stripped sequences were converted to Fasta format and merged into a single Fasta containing all sequence reads. Next, the sequences were dereplicated and their abundance determined using vsearch (ver 2.8). Denoising (error correction) was performed in Usearch (32-bit, ver 11) using the -unoise3 function with a minimum abundance of nine merged-reads per amplicon sequence variant (ASV). Taxonomy was assigned to ASVs using the 16s (V123) SILVA database with the -sintax function in usearch (32-bit, ver 11).

### Statistical Analysis

The resulting ASV abundance table was rarefied to the sample with the lowest number of reads using the R (ver 3.6) package Phyloseq (McMurdie and Holmes, 2013). The read depth was rarefied to 80,898 for each sample which was 10% less than the samples with the lowest number of reads. Rarefaction curves were plotted for each sample to ensure that the number of observed ASVs had plateaued at this read depth. The alpha diversity for each sample was calculated using four metrics; richness (total ASVs per sample), Simpson index, Shannon index, and Pielou’s evenness using the R package microbiomeSeq. Linear mixed-effects models (LMMs) were fit to the four diversity metrics using pen as a random factor in the model. The fixed effect in the model was disease stage (healthy, YM, survivor). Statistical models were fit using the “lmerTest” package in R. Assumptions of normality and homogeneity of variance were evaluated with plots of model residuals. For all statistical analyses, the significant *p*-value was set at 0.05 or less. Beta diversity analysis was performed to examine the differences between the microbial communities derived from different disease states (healthy, YM, and survivors). Bray–Curtis dissimilarity indices were calculated and visualized using non-metric multidimensional scaling (NMDS) in the package Phyloseq (McMurdie and Holmes, 2013). Multivariate homogeneity of disease group dispersions (PERMDISP2) was performed using Bray–Curtis dissimilarity indices within the R package Vegan. Statistical comparisons of the Bray–Curtis dissimilarity indices between disease groups with pen as a nested variable were performed using PERMANOVA with 999 permutations within the R package Vegan. Relative abundance plots were performed using the R package Phyloseq. To perform significance testing of taxa abundance, the ASV abundance table was first filtered to retain ASVs with an absolute abundance of >1%. This filter removed the rare low abundant taxa. Differential abundance testing at the class and ASV level was performed using DESeq2 package in R with differences between disease groups (healthy, YM, and survivor) determined using the contrast function and the Wald test. The *p*-values are corrected for multiple testing using the Benjamini and Hochberg method. Differentially abundant genera were considered significant at adjusted *p*-value < 0.01. To perform phylogenetic analysis, all ASVs classified as *T. maritimum* were aligned using MUSCLE (Edgar, 2004). This nucleotide-based multiple sequence alignment was then used to construct a phylogeny using the neighbor-joining method (Saitou and Nei, 1987).

### Microbial Networks

Microbiomes are complex microbial communities whose structure and function are strongly influenced by microbe–microbe and microbe–host interactions (Layeghifard et al., 2018). To explore these connections, we calculated pairwise correlation coefficients on relative ASV abundances for samples within each condition (healthy, YM, and survivors). ASV tables for each condition were encoded as a matrix in which each row represented a microbial taxon or ASV and each column represented an individual sample within each condition (*n* = 20). Rows with more than 2/3 zero counts (Faust et al., 2012) were filtered and aggregated into a “rare taxon group” before normalizing counts by dividing by column sums in each sample to obtain relative ASV abundances.

To build the network, we computed significant co-occurrence between rows (ASVs or microbial taxa) whose relative abundances were observed repeatedly across columns (samples). The networks were constructed with the CoNet plugin (v.1.1.1.beta) for Cytoscape v.3.7.0 (Faust and Raes, 2016) by calculating pairwise correlations between ASVs across all samples were calculated using non-parametric Spearman correlation coefficients (Faust et al., 2012). These calculations produced a matrix where microbial taxa were compared to one another in each sample, and therefore higher correlation scores indicated stronger connections between microbial pairs indicating they co-occurred more often in a given condition. Nodes in the network represented microbial taxa or ASVs and edges or links represented significant correlations. Correlations were considered significant when the Spearman correlation value ρ > 0.3 and the correlation *p*-value (corrected with Bonferroni) was *p* < 0.05.

## Results

### Sequencing Analysis

The number of raw and quality filtered merged reads generated for each sample is provided as Supplementary Table 1. On average 80% of the paired reads passed quality filtering and were successfully merged. The number of merged reads ranged between 89,887 and 257,197 per sample. A total of 4189 denoised ASVs were identified across all samples.

### Alpha and Beta Diversity

**FIGURE 1:**
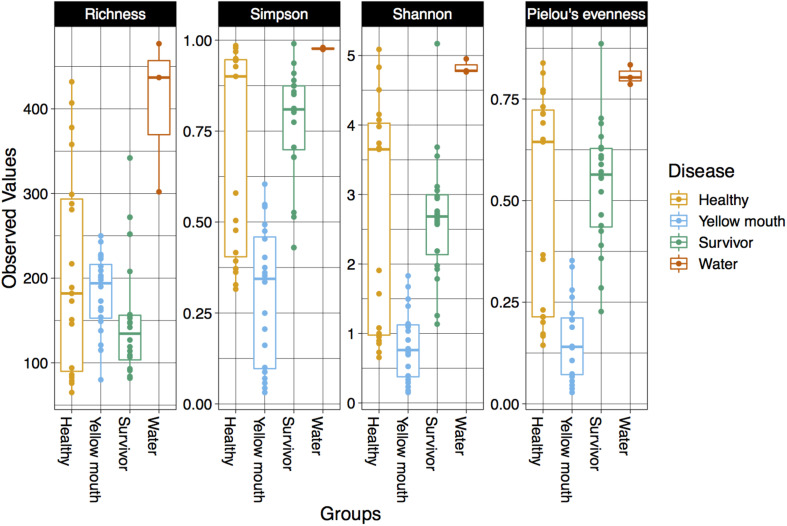
Diversity ofthe microbial community within different disease states and water samples. Boxplots show species richness, Simpsons, Shannon’s, and Pielou’s diversity indices.

The alpha diversity for each swab and water sample was calculated using four diversity indices including richness, Simpson index, Shannon index, and Pielou’s evenness. The water samples had the highest alpha diversity according to all four metrics (Figure 1). Indeed, the microbial community from the three water samples had a greater richness of ASVs, along with higher Simpson, Shannon, and Pielou’s diversity metrics compared to the swab samples. It should be noted, however, that only three water samples were collected and analyzed in this study, and while they provide some valuable information regarding the microbial community present in the water, these samples were not included in downstream statistical analysis due to the lack of replication.

Statistical comparisons were performed on all four diversity metrics between the disease groups (healthy, YM, and survivors) using a linear mixed model with “pen” as a random factor. ASV richness was not significantly different between swabs derived from healthy, YM, or surviving smolt (*p* > 0.05). However, a significant difference in the Simpson (*F*_2_,_56_ = 29.16, *p* < 0.001), Shannon (*F*_2_,_56_ = 18.42, *p* < 0.001), and Pielou’s indices (*F*_2_,_56_ = 24.55, *p* < 0.001) was observed between the disease groups. The YM samples had the lowest diversity in all three metrics (Figure 1). Taken together these results demonstrated that while the total number of ASVs remained similar in different disease states, the YM samples had a less even distribution of ASVs compared to the healthy and surviving fish. That is, they were dominated by a few highly abundant taxa.

**FIGURE 2:**
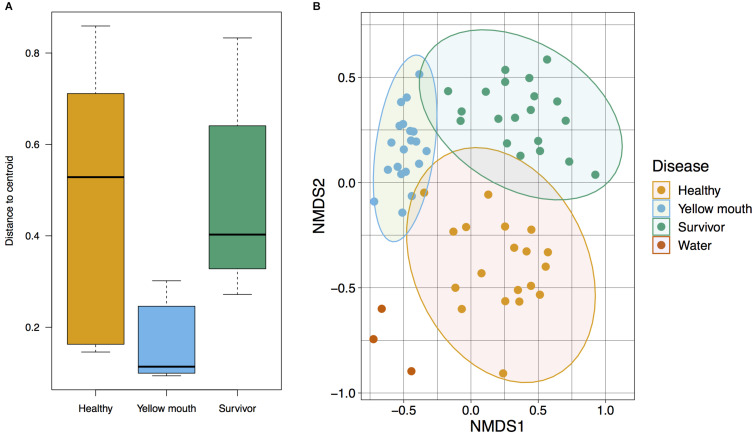
**(A)** Multivariate homogeneity of groups dispersions for Bray–Curtis distances between samples relative to the disease group centroid. **(B)** Beta diversity of microbial community derived from healthy, surviving, yellow mouth affected Atlantic salmon, and water. Non-metric dimensional scaling was performed using Bray–Curtis dissimilarity calculation (stress value = 0.20) and statistical significance testing was performed using the PERMANOVA test.

The relationship between microbial communities from the swab and water samples was assessed using a Bray–Curtis-based NMDS. Statistical significance was tested using PERMDISP and PERMANOVA analysis. Because the water samples only represented a single sample at three time points (non-replicated), they were not included in statistical analysis. PERMDISP analysis was performed to determine if the dispersions (variance) of the disease groups (healthy, YM, and survivors) were different. The distance of the disease group members to the group centroid is presented in Figure 2A; and was subjected to ANOVA. A significant difference within group variance was observed between the healthy, YM, and survivor disease groups (*F*_2_,_56_ = 15.71, *p* < 0.001). The YM samples showed less variance compared to healthy and surviving fish (Figure 2A). Due to this heterogeneity in dispersions, a PERMANOVA analysis was performed to compare the microbial communities between disease groups, as this test is largely unaffected by unequal dispersion among sampling groups (Anderson and Walsh, 2013). This analysis demonstrated a significant difference between the microbial community from the swab samples from the YM, healthy, and surviving fish (*F*_2_,_56_ = 6.95, *p* < 0.01). Compared to the healthy and surviving animals, the YM affected fish had a tighter distribution within the NMDS and PERMDISP analysis, demonstrating that the diseased fish have a largely homogenous and distinct microbial community (Figure 2B). In contrast, while the healthy and surviving fish also had distinct microbial communities, the diversity of these communities within each disease state was greater compared to the YM samples (Figure 2B).

### Taxonomic Classification

**FIGURE 3:**
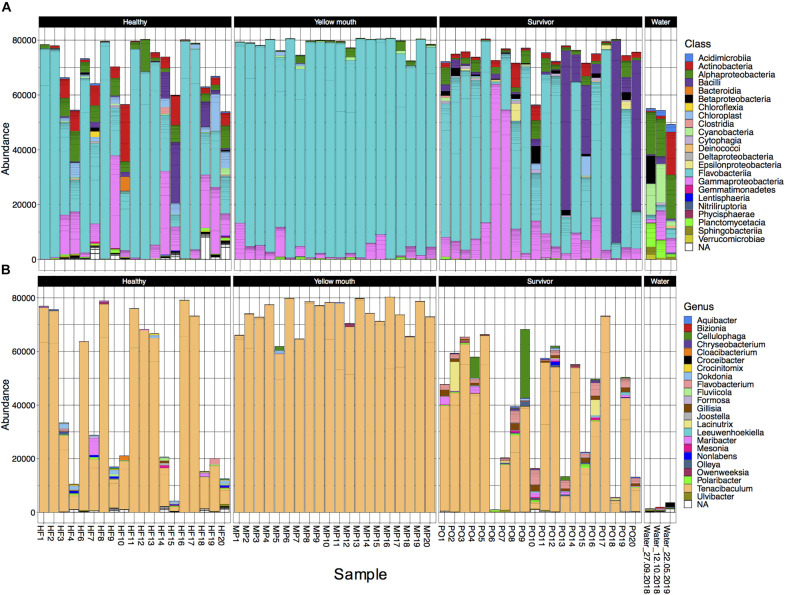
**(A)** Abundance of bacterial taxa at the “class” level from the 500 most abundant ASVs for individual animals derived from different disease states or water samples. **(B)** Abundance at the genus level for taxa classified with in the class Flavobacteria.

The 4189 ASVs were classified to 64 unique taxonomic classes (Figure 3A). The five most abundant classes in descending order of relative abundance were: Flavobacteria (mean abundance 67%), Gammaproteobacteria (12%), Bacilli (6.7%), Alphaproteobacteria (4.5%), Actinobacteria (mean 3.0%). At an individual sample level, Flavobacteria was often the most abundant class from the swab samples. Flavobacteria were observed in all disease states, albeit their relative abundance was more variable in the healthy (mean 58%, range 10–98%) and surviving (mean 53%, range 1–90%) animals. In the YM affected animals, the abundance of Flavobacteria represented 91% of the microbial community (range between 77 and 99%) (Figure 3A). At the class level, the abundance of Flavobacteria was not statistically different between the healthy, YM, and survivor groups (adjusted *p* > 0.05, df_4_,_35_). At the genus level, *Tenacibaculum* spp. was the most dominant Flavobacteria observed in the swab samples (Figure 3B). While a number of different *Tenacibaculum* species were identified, *T. maritimum* was clearly the most abundant species observed in this study (mean abundance 80%). Other *Tenacibaculum* species including *Tenacibaculum dicentrarchi* and *Tenacibaculum ovolyticum* were observed at a very low abundance (<0.1% mean abundance).

In contrast to the swab samples that were dominated by Flavobacteria, the water samples had a more even distribution across the taxa (Figure 3A), and in fact had very low abundance of *Tenacibaculum* spp. (<0.1% mean abundance). Because only a single water sample was collected at each time point, the microbial community from the water samples was not statistically compared. However, these samples did appear to show some minor changes in community composition. The abundance of Cyanobacteria and Planctomycetacia both appeared to decrease following the YM disease event, while Actinobacteria increased post-YM outbreak.

### Differential Abundance of ASVs

Statistical analysis of ASV abundance was performed between swabs derived from healthy, YM, and surviving fish as pairwise comparisons using the Wald-test in DeSeq. The abundances of 204 ASVs were significantly different between the YM and healthy fish (adjusted *p* < 0.01, df_4_,_35_, Supplementary Table 2). Similarly, the abundances of 216 ASVs were also significantly different between the surviving and healthy fish (adjusted *p* < 0.01, df_4_,_35_, Supplementary Table 2).

**FIGURE 4:**
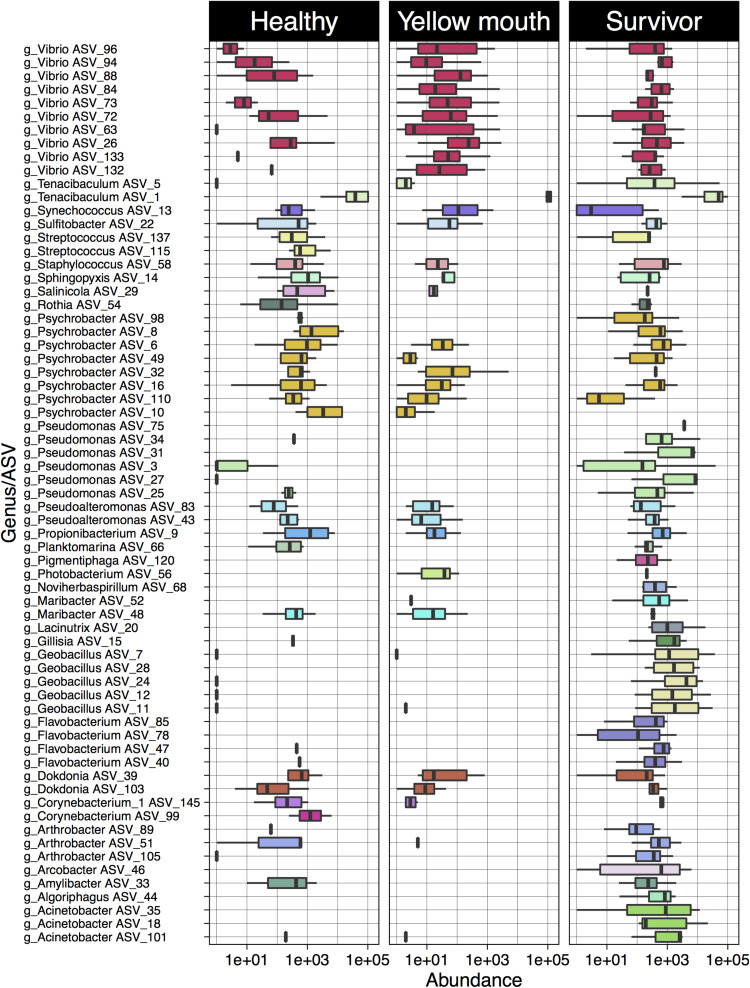
Log10 abundance for ASVs (with abundance > 1%) deemed significantly differentially abundant between the YM and healthy and/or survivor and healthy disease states. The genus level classification of each AVS is also provided.

Four ASVs classified as *T. maritimum* were significantly more abundant in the YM animals compared to healthy fish (Supplementary Table 2). In addition, a large number of *Vibrio* species were also more abundant in the YM affected fish compared to healthy fish (Figure 4). Some *Vibrio* spp. were also more abundant in the survivors compared to the healthy fish. These *Vibrio* ASVs could not be classified to the species level using the SILVA database. The healthy fish also contained a significantly higher abundance of *Streptococcus* species compared to YM and surviving fish. Multiple ASVs classified as *Geobacillus*, *Pseudomonas*, and *Acinetobacter* were found to be significantly more abundant in the surviving fish compared to the healthy animals.

### Sequence Variation in *T. maritimum* ASVs

**FIGURE 5:**
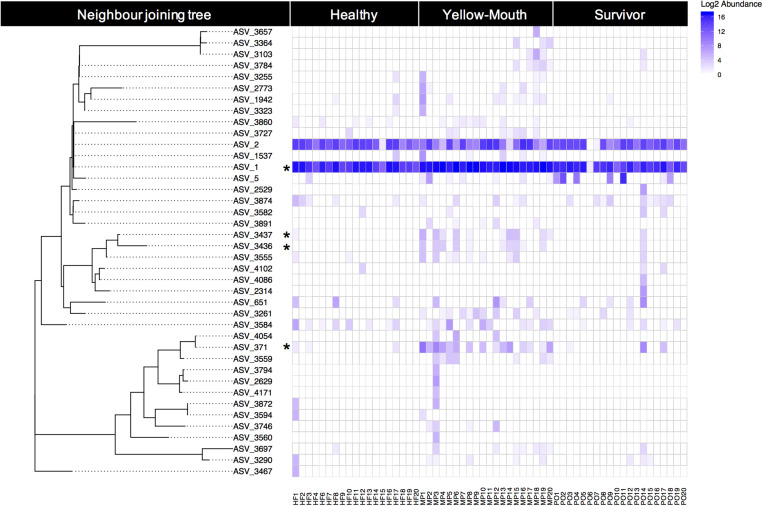
Relative (log2) abundance of ASVs that were classified as *T. maritimum* for individual fish within different disease states. The genetic relationship between ASVs was explored using a neighbor joining tree analysis and is shown on the left. *Asterisk* denotes those ASVs that were deemed significantly more abundant in the YM samples compared to healthy fish.

Over 40 ASVs were classified as *T. maritimum*. Here we examine the genetic relationship between ASVs and their individual abundances within the healthy, YM, and surviving animals. The phylogenetic relationship of the 40 ASVs was evaluated using a neighbor joining tree constructed from a multiple sequence alignment of a 491 bp region of the V1–V3 region of the 16s rRNA gene. This analysis demonstrates a close genetic relationship between ASV_1 and ASV_2, which were the most highly abundant ASVs in our dataset (Figure 5). Indeed, ASV_1 was highly abundant in almost every swab sample, with only one exception (sample PO6). As described above (Figure 4), this ASV was significantly more abundant in the YM samples compared to healthy and surviving animals (*p* < 0.001, df_4_,_35_). ASV_2 appeared less abundant, but still represented a significant proportion of the overall community. The abundance of ASV_2 was not statistically different in any disease state. In contrast to the swab samples that were dominated by *T. maritimum*, we found very low abundance of *T. maritimum* in water samples. Both ASV_1, and to a lesser extent ASV_2, were consistently the dominant ASVs in the YM samples (Figure 5). These ASVs were also highly abundant in the swab samples from the healthy and surviving fish, although their abundance was much more variable between individuals. Indeed, the abundance of ASV_1 in some healthy individuals was equivalent to the YM samples.

Using BLAST, we show that at the nucleotide level ASV_1 was 100% identical to the previously described *T. maritimum* sequence variants TmarCan1/2 (Frisch et al., 2018a) and ASV_2 was 100% identical to NLF-15 and Ch-2402 (Smage et al., 2016b; Apablaza et al., 2017). As described above and illustrated in Figure 5, ASV_371 was also significantly more abundant in the YM samples compared to healthy and surviving animals. This ASV was genetically more diverse compared to the dominant ASV_1 and ASV_2 sequence types (Figure 5). BLAST analysis demonstrated that ASV_371 has a nucleotide identity of 95% to *T. maritimum* strain TM-KORJJ.

### Microbial Networks

**FIGURE 6:**
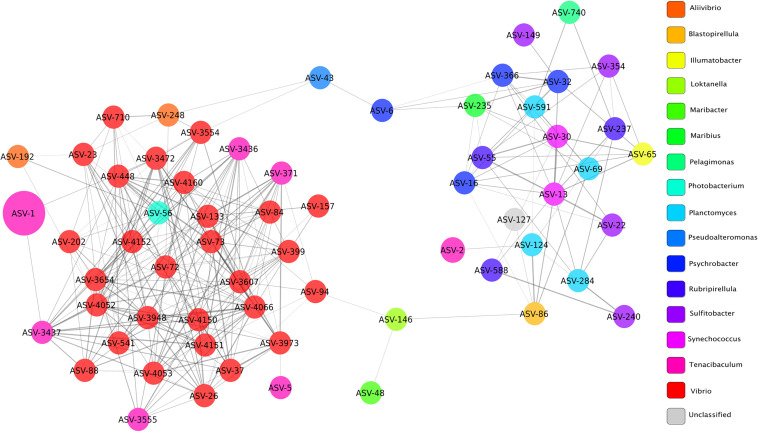
Network of bacterial taxa based on co-occurrence on all fish clinically affected by YM. Each node represents a taxon (ASV) and connections between nodes (or edges) indicate a Spearman correlation coefficient > 0.3 and a correlation *p*-value corrected with Bonferroni (<0.05). The size of each node is proportional to the relative abundance of each taxon, and the color labels indicate different genera. Edge thickness is proportional to the Spearman correlation coefficient between each node.

Microbial networks were built based on correlations of relative abundance between ASVs and therefore a network represents a set of co-occurring genera that cluster together more frequently in a given condition. Topological parameters were computed for each network (healthy, YM, and survivor) using Network Analyzer in Cytoscape (Assenov et al., 2008). The YM network (Figure 6) presented 63 nodes (ASVs) and 266 edges (significant positive correlations, ρ > 0.3 and Bonferroni corrected *p* < 0.05), all connected into one cluster with a clustering coefficient of 0.33. The clustering coefficient of a node z in a network is the probability that two nodes x and y, which are connected to the node z, are themselves connected. The average of this over all nodes z of a network is the clustering coefficient of the network. The clustering coefficient, which ranges from 0 to 1, indicates how nodes are embedded in their neighborhood and, thus, the degree to which they tend to cluster together. In microbial networks, a larger clustering coefficient indicates a more tightly connected community of microbes.

The co-occurrence network of fish with YM presented a large number of edges (65%) among ASVs classified as *Vibrio* and *Tenacibaculum*, indicating strong co-occurrence between these taxa. The node or taxon group with the highest number of connections (hub node) was ASV_4066, which belonged to the *Vibrio* genus and its occurrence was highly correlated with ASVs in the genus *Tenacibaculum*, and included ASV_317, ASV_3555, and ASV_3437. High correlation scores between ASVs in the *Vibrio* and *Tenacibaculum* genera indicate that a high relative abundance of these microbial taxa was commonly found in diseased individuals.

The co-occurrence network of healthy fish presented 28 nodes and 125 edges, with a larger clustering coefficient (0.42) than the network of YM fish (Supplementary Figure 1A). In microbial networks, a larger clustering coefficient indicates a more tightly connected community of microbes, which commonly co-occur in high abundances in healthy individuals. Most ASVs in the healthy fish network were unclassified, and ASVs classified as *Tenacibaculum* (ASV_1 and ASV_2) clustered in a separate subnetwork, which did not show any significant correlations to other taxa in the network. The co-occurrence network of survivor fish presented 31 nodes and 54 edges, with a much lower clustering coefficient (0.19) compared to the other two networks (Supplementary Figure 1B). A lower clustering coefficient suggests that the relative abundances of ASVs in surviving fish are more diverse among different individuals producing lower or non-significant correlation coefficients between different taxa, creating a more disconnected network.

## Discussion

Yellow mouth remains a major challenge for Atlantic salmon aquaculture in Western Canada and globally. The present study aimed to investigate changes in the microbial community associated with a natural YM outbreak in Atlantic salmon in Western Canada. Interestingly, while the overall number of observed taxa (ASVs) remained similar between healthy, YM, and surviving fish, the evenness (Simpson and Shannon indices) of the microbial community was significantly decreased in the YM affected animals. The oral cavity of the YM affected fish was dominated by *T. maritimum*, while other *Tenacibaculum* spp. including *T. dicentrarchi* and *T. ovolyticum* were rare. This finding is in agreement with previous work by Frisch et al. (2018a) who found that *T. maritimum* was the dominant species associated with mouth plaques in farmed Atlantic salmon in Western Canada. In contrast to the YM cases in Western Canada, outbreaks of tenacibaculosis in Norway are often dominated by a diversity of *Tenacibaculum* species, including of *Tenacibaculum finnmarkense* which clinically present as mouth erosions and frayed fins (Smage et al., 2016a; Karlsen et al., 2017). The present study did not identify any ASVs that were classified as *T. finnmarkense*. Our study demonstrates that YM is characterized by a pronounced dysbiosis of the microbial community within the oral cavity. A similar finding was within the gut microbiome of largemouth bronze gudgeon (*Coreius guichenoti*) where furunculosis affected fish had an overwhelming abundance of *Aeromonas* and a corresponding decrease in microbial diversity compared to the healthy animals (Li et al., 2016).

While *T. maritimum* was the dominate taxon in the YM affected fish, it was also highly abundant in the healthy and surviving animals. It is important to note that the “survivors” could either represent animals that became infected with YM and recovered, or animals that remained unaffected throughout the disease outbreak. Our findings highlight the multifactorial nature of YM within a commercial salmon production environment. These data indicate while the presence of the *T. maritimum* is clearly associated with YM, other factors may contribute to the development of YM in farmed Atlantic salmon in Western Canada. Previous studies have also found that Flavobacteriacea, including members of the *Tenacibaculum* spp., are often associated with healthy skin of farmed Atlantic salmon (Karlsen et al., 2017). Our study suggests that a proportion of the fish deemed healthy, were either sub-clinically or pre-clinically infected with *T. maritimum* yet showed no clinical signs of YM. Similarly, Li et al. (2016) reported that healthy (non-furunculosis affected) fish also had reasonable high abundance of *Aeromonas*, albeit less than the fish clinically affected by furunculosis. Given that our study did not perform follow-up sampling of these healthy fish, the progression to clinical disease following sampling cannot be ruled out. The surviving fish were sampled in November after mortality had resolved and showed no clinical signs of disease, yet some individuals had a high abundance of *T. maritimum*. Taken together we conclude that while *T. maritimum* is clearly associated with YM, additional factors may contribute to clinical disease.

The ability of *T. maritimum* to dominate the microbial population even in the healthy and surviving animals suggests this species is highly adapted to the ecological niche it occupies and may in fact be part of the normal commensal microbial community of Atlantic salmon smolt. The ability of *T. maritimum* to opportunistically trigger disease may, in part, be due to reduced immune function associated with external stress events (such as smoltification or husbandry operations). While the ability of *T. maritimum* to either passively or actively inhibit the growth of neighboring taxa was not examined here, a growing body of evidence suggests that some members of the Tenacibaculum genus can competitively inhibit both Gram-negative and -positive taxa. Indeed, studies on the *Tenacibaculum* sp. strain 20J have demonstrated tha this species contains a novel acyl homoserine lactone (AHL) that has broad-spectrum quorum quenching activity against virulent *Escherichia coli* (Mayer et al., 2015). Purified or crude cell extracts from the same species were also able to reduce biofilm formation of *Streptococcus mutans* (Muras et al., 2018) and inhibit the production of AHL by the fish pathogen *Edwardsiella tarda* (Romero et al., 2014). The ability of the dominant *T. maritimum* strains described herein to competitively inhibit other taxa within the oral cavity requires further targeted investigation. In this context, it is important to note that not all *Tenacibaculum* strains have been shown to process quorum quenching activity (Romero et al., 2010). Furthermore, the possibility that *T. maritimum* dominates the microbial community through a passive competitive exclusion whereby they either restrict competitive access to nutrients or space should also not be excluded.

Network analysis was used to identify co-occurrence between ASVs within the different disease states. We found that the abundances of many *Vibrio* spp. were significantly associated with the abundance of *T. maritimum* in the YM affected fish. A significant co-occurrence between *Tenacibaculum* and *Vibrio* was only observed in the animals displaying clinical signs of YM. Furthermore, we observed a significant increase in *Vibrio* abundance in YM affected animals compared to the healthy fish. These findings suggest that *Vibrio* may play a role in YM; however, the nature of this relationship and any role *Vibrio* plays in clinical manifestation of YM remains unclear and needs to be explored further. We speculate that *Vibrio* may act as an opportunistic pathogen that colonizes the compromised tissue following a primary *T. maritimum* infection. Mixed infection of *Tenacibaculum* and *Vibrio* has been reported in a number of marine species previously. Indeed, in Tasmania (Australia), co-isolation of *T. maritimum* along with mixed *Vibrio* spp. was reported from Atlantic salmon skin lesions (Handlinger et al., 1997). Given that we found an increase in *Vibrio* abundance associated with clinical MR, it is possible that *Vibrio* may exacerbate lesion pathology and thus enhance the severity of clinical disease. Previous studies have shown that co-infection with *Vibrio* sp. can synergistically increase disease severity in the context of both viral, bacterial, and parasite infections (Kotob et al., 2016). Indeed, Atlantic salmon co-challenged with infectious pancreatic necrosis virus (IPNV) and *Vibrio salmonicida* showed increased mortality compared to animals challenged with the bacterium alone (Johansen and Sommer, 2001). Furthermore, *Vibrio* sp. have been shown to affect many aquatic species as either the primary or opportunistic pathogen (Austin and Austin, 2007). Based on our 16s rRNA amplicon sequencing analysis, we were not able to classify many of the *Vibrio* ASVs down to the species level. Further research is required to determine the role that these *Vibrio* species may play in YM disease.

We identified a number of genetically distinct ASVs that were classified as *T. maritimum*. While individual fish appeared to harbor a diversity of *T. maritimum* sequence variants, all fish (regardless of disease status) were dominated by two ASVs. The most abundant two ASVs (ASV_1 and ASV_2) were 100% identical to previously isolated *T. maritimum* strains from both Canada and Norway. Indeed ASV_1 was identical to the Tmar_Can1/2 genotypes previously isolated and sequenced from mouthrot affected Atlantic salmon in BC (Frisch et al., 2018a). These genotypes were found to be the dominant strain types in Western Canada and were closely related to Norwegian and Chilean derived isolates (Frisch et al., 2018a). Given that our sequenced amplicon only covers the first to third variable regions of 16s, our study was unable to discriminate between the Tmar_Can1 and Tmar_Can2 sequence types which were 100% identical across this region (Frisch et al., 2018a). With this in mind, it is highly likely that additional variation exists within the dominant ASV_1 sequence variant. The second most abundant taxa, ASV_2, was 100% identical to the NLF-15 sequence variant isolated from lumpsucker (*Cyclopterus lumpus*) from Norway (Smage et al., 2016b), but also 100% identical to the Chilean Atlantic salmon derived strain, Ch-2402, (Apablaza et al., 2017). Some less abundant *T. maritimum* ASVs appeared to be significantly more abundant in the YM affected fish including ASV_371 related to Korean isotypes TM-KORJJ (GenBank accession CP020822.1). This finding suggests that some *Tenacibaculum* classified ASVs are more closely related to the disease phenotype than others, potentially reflecting differences in virulence between genetic isotypes.

Non-metric multidimensional scaling revealed clear separation among the disease status groups and water samples. Interestingly the healthy and surviving groups showed more individual fish to fish variation compared to the YM group. This finding suggests that at a population level the microbiome of healthy animals maybe quite diverse, possibly reflecting genetic, behavioral, or environmental influences. The microbial community of the water samples was significantly more diverse and appeared distinct to that derived from the fish. This finding agrees with other studies on Atlantic salmon where the microbial community on the skin (either healthy or ulcer affected) was significantly different to the bacterial community in the water column (Karlsen et al., 2017). Interestingly, the water samples collected pre-stocking, during, and after the YM outbreak had a relatively low abundance of *Tenacibaculum*. This finding is in agreement with previous studies that demonstrate a poor survival of *T. maritimum* under natural seawater conditions (Avendano-Herrera et al., 2006a). We found no significant increase in *Tenacibaculum* abundance in the water during the outbreak compared to pre-stocking or post-outbreak. Given our study specifically examined *Tenacibaculum* in the water column, it is possible that *Tenacibaculum* abundance may have increased, however been localized to the biofilm on the pen infrastructure (i.e., nets, pens, ropes) or in the sediment under the pen. Recent studies have also shown that jellyfish may play a role in *T. maritimum* infection and transmission. Indeed, Ferguson et al. (2010) demonstrated that *T. maritimum* was capable of causing a secondary infection in the gills of Atlantic salmon following a primary gill injury caused by jellyfish (*Phialella quadrata*) derived nematocysts. The authors went on to show that *T. maritimum* was also present on the jellyfish and subsequently concluded that jellyfish may act as a vector capable of transmitting this bacterium. Regardless, it is clear that *Tenacibaculum* is present in the farm environment before fish are stocked, and the development of YM can occur without a significant increase in *Tenacibaculum* in the water column.

## Conclusion

Using a 16s rRNA microbiome analysis, we demonstrate a significant reduction in microbial diversity in the YM affected animals with the community dominated by two genetic variants of *T. maritimum*. Interestingly, clinically unaffected healthy and YM surviving animals also had high relative abundance of *T. maritimum*, suggesting that the presence of *T. maritimum* is not solely responsible for YM. A statistically significant association was observed between YM and increased abundance of *Vibrio* spp. around the mouth lesion. Taken together our study provides further evidence that YM is a multifactorial disease, and while dominated by diverse isotypes of *T. maritimum* other commensal taxa, including *Vibrio* spp., may also play a role in clinical disease progression.

## Data Availability Statement

The datasets presented in this study can be found in online repositories. The names of the repository/repositories and accession number(s) can be found below: https://www.ncbi.nlm.nih.gov/, PRJNA647328.

## Ethics Statement

Ethical review and approval was not required for the animal study because this study utilized swab samples that had been collected during a veterinary investigation of a natural yellow mouth disease event in British Columbia. As these samples were collected on the farm during a routine disease investigation, no animal ethics approval was required.

## Author Contributions

AS, BM, JP, and AM prepared the original project concept. AS, KT, IG, ON, BM, AM, and JW formulated the study design and project objectives. DN provided samples and production data. ZR performed nucleic acid extractions and QC. JW, JS, and FS performed bioinformatic analysis. JW, AS, and KT performed data interpretation. JW, KT, and AS wrote the manuscript, with input from all co-authors. All authors contributed to the article and approved the submitted version.

## Conflict of Interest

The authors declare that the research was conducted in the absence of any commercial or financial relationships that could be construed as a potential conflict of interest.

## Supplementary Material

Supplementary Figure 1 Network of bacterial taxa based on co-occurrence on all healthy fish **(A)** and surviving fish **(B)**. Each node represents a taxon (ASV) and connections between nodes (or edges) indicate a Spearman correlation coefficient > 0.3 and a correlation *p*-value corrected with Bonferroni (<0.05). The size of each node is proportional to the relative abundance of each taxon, and the color labels indicate different genera. Edge thickness is proportional to the Spearman correlation coefficient between each node.

Supplementary Table 1 Raw and merged sequence output statistics for swab and water samples processed in this study.

Supplementary Table 2 Pairwise differential abundance testing statistics at the ASV level between healthy, yellow mouth, and survivor groups. Only ASVs that were significantly differentially abundant (adjusted *p* < 0.01) are illustrated here.

## References

[B1] Anderson M. J. Walsh D. C. I. (2013). PERMANOVA, ANOSIM, and the Mantel test in the face of heterogeneous dispersions: what null hypothesis are you testing? *Ecol. Monogr.* 83 557–574. 10.1890/12-2010.1

[B2] Apablaza P. Frisch K. Brevik O. J. Smage S. B. Vallestad C. Duesund H. (2017). Primary isolation and characterization of *Tenacibaculum maritimum* from Chilean Atlantic salmon mortalities associated with a *Pseudochattonella* spp. algal bloom. *J. Aquat. Anim. Health* 29 143–149. 10.1080/08997659.2017.1339643 28613984

[B3] Austin B. Austin D. (2007). *Bacterial Fish Pathogens: Diseases of Farmed and Wild Fish.* Berlin: Springer.

[B4] Avendano-Herrera R. Irgang R. Magarinos B. Romalde J. L. Toranzo A. E. (2006a). Use of microcosms to determine the survival of the fish pathogen *Tenacibaculum maritimum* in seawater. *Environ. Microbiol.* 8 921–928. 10.1111/j.1462-2920.2005.00981.x 16623748

[B5] Avendano-Herrera R. Toranzo A. E. Magarinos B. (2006b). Tenacibaculosis infection in marine fish caused by *Tenacibaculum maritimum*: a review. *Dis. Aquat. Organ* 71 255–266. 10.3354/dao071255 17058606

[B6] Assenov Y. Ramirez F. Schelhorn S. E. Lengauer T. Albrecht M. (2008). Computing topological parameters of biological networks. *Bioinformatics* 24, 282–284. 10.1093/bioinformatics/btm554 18006545

[B7] Edgar R. C. (2004). MUSCLE: multiple sequence alignment with high accuracy and high throughput. *Nucleic Acids Res.* 32 1792–1797. 10.1093/nar/gkh340 15034147 PMC390337

[B8] Egan S. Gardiner M. (2016). Microbial dysbiosis: rethinking disease in marine ecosystems. *Front. Microbiol.* 7:991. 10.3389/fmicb.2016.00991 27446031 PMC4914501

[B9] Faust K. Raes J. (2016). CoNet app: inference of biological association networks using Cytoscape. *F1000Res.* 5:1519. 10.12688/f1000research.9050.2 27853510 PMC5089131

[B10] Faust K. Sathirapongsasuti J. F. Izard J. Segata N. Gevers D. Raes J. (2012). Microbial co-occurrence relationships in the human microbiome. *PLoS Comput Biol.* 8:e1002606. 10.1371/journal.pcbi.1002606 22807668 PMC3395616

[B11] Ferguson H. W. Delannoy C. M. J. Hay S. Nicolson J. Sutherland D. Crumlish M. (2010). Jellyfish as vectors of bacterial disease for farmed salmon (*Salmo salar*). *J. Vet. Diagn. Invest.* 22 376–382. 10.1177/104063871002200305 20453210

[B12] Fernandez-Alvarez C. Santos Y. (2018). Identification and typing of fish pathogenic species of the genus Tenacibaculum. *Appl. Microbiol. Biot.* 102 9973–9989. 10.1007/s00253-018-9370-1 30291367

[B13] Frisch K. Smage S. B. Brevik O. J. Duesund H. Nylund A. (2018a). Genotyping of *Tenacibaculum maritimum* isolates from farmed Atlantic salmon in Western Canada. *J. Fish Dis.* 41 131–137. 10.1111/jfd.12687 28744871

[B14] Frisch K. Smage S. B. Vallestad C. Duesund H. Brevik O. J. Klevan A. (2018b). Experimental induction of mouthrot in Atlantic salmon smolts using *Tenacibaculum maritimum* from Western Canada. *J. Fish Dis.* 41 1247–1258. 10.1111/jfd.12818 29761493

[B15] Habib C. Houel A. Lunazzi A. Bernardet J. F. Olsen A. B. Nilsen H. (2014). Multilocus sequence analysis of the marine bacterial genus *Tenacibaculum* suggests parallel evolution of fish pathogenicity and endemic colonization of aquaculture systems. *Appl. Environ. Microb.* 80 5503–5514. 10.1128/aem.01177-14 24973065 PMC4136090

[B16] Handlinger J. Soltani M. Percival S. (1997). The pathology of *Flexibacter maritimus* in aquaculture species in Tasmania, Australia. *J. Fish Dis.* 20 159–168. 10.1046/j.1365-2761.1997.00288.x

[B17] Johansen L. H. Sommer A. I. (2001). Infectious pancreatic necrosis virus infection in Atlantic salmon *Salmo salar* post-smolts affects the outcome of secondary infections with infectious salmon anaemia virus or *Vibrio salmonicida*. *Dis. Aquat. Org.* 47 109–117. 10.3354/dao047109 11775792

[B18] Karlsen C. Ottem K. F. Brevik O. J. Davey M. Sorum H. Winther-Larsen H. C. (2017). The environmental and host-associated bacterial microbiota of Arctic seawater-farmed Atlantic salmon with ulcerative disorders. *J. Fish Dis.* 40 1645–1663. 10.1111/jfd.12632 28449237

[B19] Kotob M. H. Menanteau-Ledouble S. Kumar G. Abdelzaher M. El-Matbouli M. (2016). The impact of co-infections on fish: a review. *Vet. Res.* 47:98. 10.1186/s13567-016-0383-4 PMC5050641 27716438

[B20] Lane D. (1991). *Nucleic Acid Techniques in Bacterial Systematics.* New York, NY: Wiley.

[B21] Lane D. J. Pace B. Olsen G. J. Stahl D. A. Sogin M. L. Pace N. R. (1985). Rapid-determination of 16s ribosomal-RNA sequences for phylogenetic analyses. *Proc. Natl. Acad. Sci. U.S.A.* 82 6955–6959. 10.1073/pnas.82.20.6955 2413450 PMC391288

[B22] Layeghifard M. Hwang D. M. Guttman D. S. (2018). *Constructing and Analyzing Microbiome Networks in R.* New York, NY: Humana Press. 10.1007/978-1-4939-8728-3_16 30298259

[B23] Li T. T. Long M. Ji C. Shen Z. X. Gatesoupe F. J. Zhang X. J. (2016). Alterations of the gut microbiome of largemouth bronze gudgeon (*Coreius guichenoti*) suffering from furunculosis. *Sci. Rep.* 6:30606. 10.1038/srep30606 PMC4964610 27465687

[B24] Mayer C. Romero M. Muras A. Otero A. (2015). Aii20J, a wide-spectrum thermostable N-acylhomoserine lactonase from the marine bacterium *Tenacibaculum* sp. 20J, can quench AHL-mediated acid resistance in *Escherichia coli*. *Appl. Microbiol. Biotechnol.* 99 9523–9539. 10.1007/s00253-015-6741-8 26092757

[B25] McMurdie P. J. Holmes S. (2013). Phyloseq: an R package for reproducible interactive analysis and graphics of microbiome census data. *PLoS One* 8:e61217. 10.1371/journal.pone.0061217 23630581 PMC3632530

[B26] Muras A. Mayer C. Romero M. Camino T. Ferrer M. D. Mira A. (2018). Inhibition of *Steptococcus* mutans biofilm formation by extracts of *Tenacibaculum* sp. 20J, a bacterium with wide-spectrum quorum quenching activity. *J. Oral. Microbiol.* 10:1429788. 10.1080/20002297.2018.1429788 29410771 PMC5795696

[B27] Powell J. Podlasly T. (2015). *Tenacibaculum Maritimum: Current Knowledge and Future Directions.* Campbell River: CAHS workshop.

[B28] Romero M. Avendano-Herrera R. Magarinos B. Camara M. Otero A. (2010). Acylhomoserine lactone production and degradation by the fish pathogen *Tenacibaculum maritimum*, a member of the *Cytophaga*-Flavobacterium-*Bacteroides* (CFB) group. *FEMS Microbiol Lett.* 304 131–139. 10.1111/j.1574-6968.2009.01889.x 20377642

[B29] Romero M. Muras A. Mayer C. Bujan N. Magarinos B. Otero A. (2014). *In vitro* quenching of fish pathogen *Edwardsiella tarda* AHL production using marine bacterium *Tenacibaculum* sp. strain 20J cell extracts. *Dis. Aquat. Organ.* 108 217–225. 10.3354/dao02697 24695235

[B30] Saitou N. Nei M. (1987). The neighbor-joining method–a new method for reconstructing phylogenetic trees. *Mol. Biol. Evol.* 4 406–425. 3447015 10.1093/oxfordjournals.molbev.a040454

[B31] Simon C. J. Truong H. H. Noble T. H. Osborne S. A. Wynne J. W. Wade N. M. (2020). Microbial biomass, marine invertebrate meals and feed restriction influence the biological and gut microbiota response of shrimp *Penaeus monodon*. *Aquaculture* 520:734679 10.1016/j.aquaculture.2019.734679

[B32] Smage S. B. Brevik O. J. Duesund H. Ottem K. F. Watanabe K. Nylund A. (2016a). *Tenacibaculum finnmarkense* sp. nov., a fish pathogenic bacterium of the family Flavobacteriaceae isolated from Atlantic salmon. *Antonie Van Leeuwenhoek* 109 273–285. 10.1007/s10482-015-0630-0 26662517 PMC4751178

[B33] Smage S. B. Frisch K. Brevik O. J. Watanabe K. Nylund A. (2016b). First isolation, identification and characterisation of *Tenacibaculum maritimumin* Norway, isolated from diseased farmed sea lice cleaner fish *Cyclopterus lumpus* L. *Aquaculture* 464 178–184. 10.1016/j.aquaculture.2016.06.030

