# Supplementary material for: Microbiome Profiling Reveals a Microbial Dysbiosis During a Natural Outbreak of Tenacibaculosis (Yellow Mouth) in Atlantic Salmon
Source: Front Microbiol. 2020 Oct 22;11:586387. doi: 10.3389/fmicb.2020.586387 (PMC7642216; doi:10.3389/fmicb.2020.586387)
Supplement: Supplementary Figure 1 — Network of bacterial taxa based on co-occurrence on all healthy fish (A) and surviving fish (B). Each node represents a taxon (ASV) and connections between nodes (or edges) indicate a Spearman correlation coefficient > 0.3 and a correlation p-value corrected with Bonferroni (<0.05). The size of each node is proportional to the relative abundance of each taxon, and the color labels indicate different genera. Edge thickness is proportional to the Spearman correlation coefficient between each node. [file Image_1.pdf]

Supplementary Material

1.1 Supplementary Figures

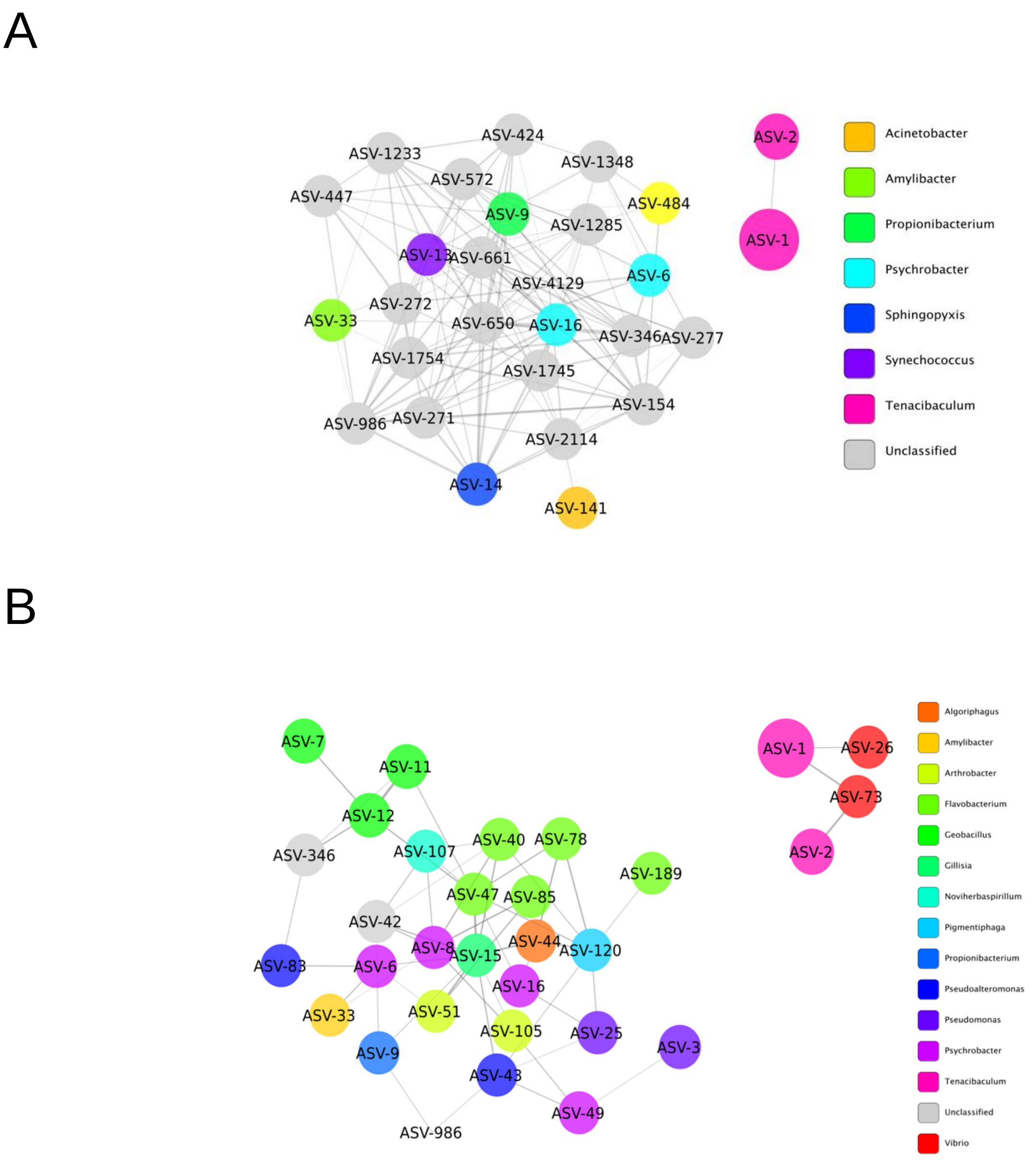

Supplementary Figure 1. Network of bacterial taxa based on co-occurrence on all healthy fish (A) and surviving fish (B). Each node represents a taxon (ASV) and connections between nodes (or edges) indicate a Spearman correlation coefficient > 0.3 and a correlation p-value corrected with

Bonferroni (<0.05). The size of each node is proportional to the relative abundance of each taxon, and the colour labels indicate different genera. Edge thickness is proportional to the Spearman correlation coefficient between each node.
